# Developmental regulation of cellular metabolism is required for intestinal elongation and rotation

**DOI:** 10.1242/dev.202020

**Published:** 2024-02-19

**Authors:** Julia K. Grzymkowski, Yu-Chun Chiu, Dereje D. Jima, Brent H. Wyatt, Sudhish Jayachandran, Whitney L. Stutts, Nanette M. Nascone-Yoder

**Affiliations:** ^1^ Department of Molecular Biomedical Sciences, College of Veterinary Medicine, North Carolina State University, Raleigh, NC 27607, USA; ^2^ Molecular Education, Technology and Research Innovation Center (METRIC), Raleigh, NC 27695, USA; ^3^ Center for Human Health and the Environment, North Carolina State University, Raleigh, North Carolina 27695, USA; ^4^ Bioinformatics Research Center, North Carolina State University, Raleigh, NC 27607, USA; ^5^ Department of Molecular and Structural Biochemistry, North Carolina State University, Raleigh, NC 27695, USA

**Keywords:** Intestine, Metabolism, Rotation, Electron transport chain, Elongation, Atrazine, *Xenopus*

## Abstract

Malrotation of the intestine is a prevalent birth anomaly, the etiology of which remains poorly understood. Here, we show that late-stage exposure of *Xenopus* embryos to atrazine, a widely used herbicide that targets electron transport chain (ETC) reactions, elicits intestinal malrotation at high frequency. Interestingly, atrazine specifically inhibits the cellular morphogenetic events required for gut tube elongation, including cell rearrangement, differentiation and proliferation; insufficient gut lengthening consequently reorients the direction of intestine rotation. Transcriptome analyses of atrazine-exposed intestines reveal misexpression of genes associated with glycolysis and oxidative stress, and metabolomics shows that atrazine depletes key glycolytic and tricarboxylic acid cycle metabolites. Moreover, cellular bioenergetics assays indicate that atrazine blocks a crucial developmental transition from glycolytic ATP production toward oxidative phosphorylation. Atrazine-induced defects are phenocopied by rotenone, a known ETC Complex I inhibitor, accompanied by elevated reactive oxygen species, and rescued by antioxidant supplementation, suggesting that malrotation may be at least partly attributable to redox imbalance. These studies reveal roles for metabolism in gut morphogenesis and implicate defective gut tube elongation and/or metabolic perturbations in the etiology of intestinal malrotation.

## Teaser

**Highlighted Article:** Late-stage exposure to atrazine disrupts cellular metabolism in the developing gut tube, leading to transcriptional, cellular and metabolic changes that perturb the proper elongation and rotation of the intestine.

## INTRODUCTION

During digestive system development, the vertebrate gut tube lengthens dramatically and undergoes species-specific convolutions to correctly package the intestine inside the body cavity. In humans, this process is known as intestinal rotation and is classically believed to be driven by a ∼270° revolution of the embryonic intestinal tract (Grzymkowski et al., 2020). It has been suggested, however, that the rotation process may not involve a literal, *en masse* revolution of the entire intestine. Instead, the final anatomy may result from sequential looping of more limited regions of the lengthening tract, which merely lends the appearance of the whole intestine having executed a massive rotation (Soffers et al., 2015; Grzymkowski et al., 2020). Thus, despite decades of investigation, the process of intestine rotation remains controversial and poorly understood.

In as many as 1 in 500 human births, the gut tube fails to properly execute rotation, leading to intestinal malrotation (IM), one of the most common structural birth anomalies (Torres and Ziegler, 1993; McVay et al., 2007; Martin and Shaw-Smith, 2010). IM phenotypes are highly variable, ranging from reversed rotation to incomplete and non-rotation, with both typical and atypical configurations (Mehall et al., 2002; McVay et al., 2007; Dassinger and Smith, 2012). In individuals with IM, segments of the developing intestine that normally are located within specific left or right quadrants of the abdomen become repositioned and/or improperly fixed, predisposing the gut tube to volvulus, a life-threatening condition requiring surgical intervention, usually within the first year of life (Ezer et al., 2016).

Despite the prevalence of IM, knowledge of its etiology is scant, partially owing to the complexity and limited understanding of the rotation process. Although IM phenotypes are associated with abnormal left–right asymmetry (Ticho et al., 2000; Shiraishi and Ichikawa, 2012; Kothari, 2014), consistent with the inherent chirality of intestine rotation, most cases occur sporadically and independently of left–right anomalies. Thus, few genetic associations have been identified to help explain the underlying molecular or cellular pathophysiology of isolated or non-laterality-related malrotation (Martin and Shaw-Smith, 2010; Salehi Karlslätt et al., 2019).

The high incidence of IM suggests environmental exposures may also contribute to these anatomical anomalies (Zhaurova, 2008; Gilbert-Barness, 2010; Krauss and Hong, 2016). Recently, the widely used herbicide atrazine (ATR) was reported to perturb gut morphogenesis in developing tadpoles (Lenkowski et al., 2008; Rutkoski et al., 2018). Interestingly, ATR was found to elicit intestine rotation anomalies when applied at late organogenesis stages, i.e. subsequent to left–right patterning events, but the mechanism by which ATR affects gut morphogenesis has not been determined. As a weed-killing herbicide, ATR was formulated to inhibit photosynthesis by blocking the electron transport chain (ETC) of chloroplast Photosystem II (Steinback et al., 1981; Dan Hess, 2000; Rutherford and Krieger-Liszkay, 2001). Importantly, structural and functional similarities between photosynthetic and mitochondrial ETC componentry suggest that ATR could also inhibit mitochondrial electron transport, thus crippling oxidative phosphorylation (OXPHOS) in animal cells. Indeed, in adult animals and animal cell culture, exposure to ATR has been shown to cause oxidative stress and metabolic dysfunction consistent with its inhibition of mitochondrial ETC Complex I (Lim et al., 2009; Zaya et al., 2011; Jin et al., 2014; Semren et al., 2018; Yin et al., 2020; Jiang et al., 2021; Karadayian et al., 2023). Unfortunately, whether or how ATR affects cellular metabolic states in the developing embryo, and the potential consequences of metabolic perturbations for vertebrate gut morphogenesis, are largely unknown.

Here, we show that late-stage exposure of *Xenopus* embryos to ATR perturbs the cellular morphogenetic events necessary for sufficient gut elongation, including endoderm cell rearrangement, differentiation and proliferation, resulting in anatomical displacement and reversal of intestine coiling. Transcriptomic, metabolomic and metabolic analyses indicate that ATR exposure inhibits ETC Complex I in embryonic gut cells, increasing oxidative stress and blocking the developmental transition from a largely glycolytic metabolism to OXPHOS. Remarkably, both elongation and rotation can be rescued by pre-treatment with an antioxidant, suggesting that ATR-induced malrotation is at least partly attributable to redox imbalance. Taken together, our results reveal roles for cellular metabolic pathways in gut elongation and rotation, with implications for the potential contribution of metabolic insults and/or inadequate gut lengthening to the etiology of IM.

## RESULTS

### Developmental exposure to atrazine causes intestinal malrotation

**Fig. 1.**
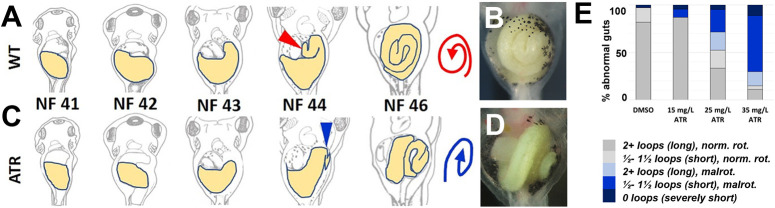
**Exposure to atrazine causes intestinal shortening and malrotation.** (A,C) Schematics illustrating normal counterclockwise (CCW) intestine rotation in wild-type (WT) *Xenopus* embryos (A), and the abnormal clockwise (CW) malrotation seen in ATR-exposed embryos (C). The midgut (future intestine) is yellow. Red (WT) and blue (ATR) arrowheads indicate the intestinal apex (NF 44, establishes the initial direction of rotation), and red (WT) and blue (ATR) spirals illustrate the final rotation direction of the intestinal coil (NF 46). (B,D) *In situ* stereo-microscope images of DMSO or ATR-treated NF 46 intestines (ventral view). DMSO control embryos develop elongated intestines that rotate normally (B), whereas ATR-exposed embryos develop intestine coils that are both short and malrotated (D). (E) The frequency of abnormal gut phenotypes increases with increasing concentrations of ATR, from predominantly normal (norm.) length (2+ intestine loops) and CCW rotation (rot.) to increasingly short (1.5 or fewer intestine loops) and/or CW malrotated (malrot.) configurations.

As observed in other vertebrates, the *Xenopus* intestine lengthens substantially (∼3-fold) during pre-feeding gut morphogenesis, i.e. Nieuwkoop and Faber (NF) stage 41-46 (Zahn et al., 2022; Fig. 1A). To package this excessive length inside the relatively small body cavity, the gut tube is tightly wound into a compact intestinal coil, rotated counterclockwise (CCW) (Chalmers and Slack, 1998; Muller et al., 2003; Zahn et al., 2022). The process that orients this configuration is most evident at NF 44, when the midgut–hindgut boundary of the gut tube (situated in the anterior-left quadrant of the visceral cavity) forms a hairpin loop that turns sharply posterior and comes to lie ventral to the midgut (Fig. 1A, NF 44, red arrowhead). As the midgut and hindgut lengthen, this initial hairpin loop is retained and will become the apex (i.e. center) of the coil, impelling the elongating intestine to rotate around it in a CCW direction as concentric loops of gut are continuously added to the outside of the coil (Fig. 1A, NF 46, red spiral arrow).

To clarify exactly how ATR affects rotation, we exposed *Xenopus* embryos to varying concentrations of ATR just prior to and during intestine morphogenesis (NF 39-46). In contrast to the CCW-rotated intestine coil of DMSO controls (Fig. 1A,B), the intestine of ATR-exposed embryos formed the hairpin loop in a different position, i.e. more dorsally and/or at a different angle (e.g. in a more sagittal plane; Fig. 1C, NF 44, blue arrowhead). This displacement of the apex resulted in a clockwise (CW) rotated coil, visible on the left side of the embryo (Fig. 1C,D, NF 46, blue spiral arrow). The frequency of such malrotations increased with higher concentrations of ATR, and was associated with shortening of the gut tube, as indicated by its more direct (less curved) route across the body (compare NF 43-44 in Fig. 1A and C) and, ultimately, fewer loops of intestine comprising the malrotated coil (Fig. 1E; 26.5% shorter, on average, *P*<0.01).

To assess whether ATR-induced malrotation may be attributed to the known effect of ATR on electron transport, we exposed embryos to a second herbicide that also inhibits chloroplast Photosystem II (Diuron). Interestingly, despite having a different chemical structure, Diuron precisely phenocopied the distinctive malrotation defects induced by ATR (Fig. S1), suggesting the two compounds may exert a common metabolic effect on gut morphogenesis.

Although intestines exposed to ATR/Diuron were shorter, i.e. similar in length to earlier stages, the embryos were not merely developmentally delayed as their craniofacial features, overall body length and accessory digestive organs continued to develop at the same pace as that observed in DMSO controls. Interestingly, however, the abnormal gut morphogenesis in ATR embryos was at least partly reversible, as removing the ATR after initial exposure restored both gut lengthening and normal rotation if performed prior to the formation of the hairpin loop (as late as NF 42-43; Fig. S2). These observations suggest that the final direction of intestine rotation is not determined until relatively late in gut morphogenesis and is linked to the degree of gut elongation.

### ATR inhibits intestinal cell mesenchymal-to-epithelial transition

We next investigated the phenotypes caused by ATR exposure at the cellular level. The association of intestinal malrotation with shorter gut lengths in ATR-exposed embryos suggested that the underlying morphogenetic processes that drive gut elongation are perturbed by ATR. To test this hypothesis, we compared elongation-related cellular properties in DMSO- and ATR-exposed guts.

Prior to lengthening, the *Xenopus* gut tube is filled with several layers of undifferentiated endoderm cells (i.e. the future intestinal epithelium) with mesenchymal characteristics. During early gut elongation (NF 40-43; see Fig. 1A), these cells undergo an intercalation process in which they gradually become radially polarized, i.e. like the spokes of a wheel, and move toward the basement membrane to thin the nascent gut epithelium into a single (pseudostratified) layer. These rearrangements are thought to simultaneously convert gut width to length, impelling the elongating midgut to curve along the body wall and become oriented transversely. Concomitant with rearrangement, the cells undergo a mesenchymal-to-epithelial transition (MET), transforming the layers of undifferentiated endoderm into intestinal epithelium (Chalmers and Slack, 2000; Reed et al., 2009; Dush and Nascone-Yoder, 2013; Pickett et al., 2017).

**Fig. 2.**
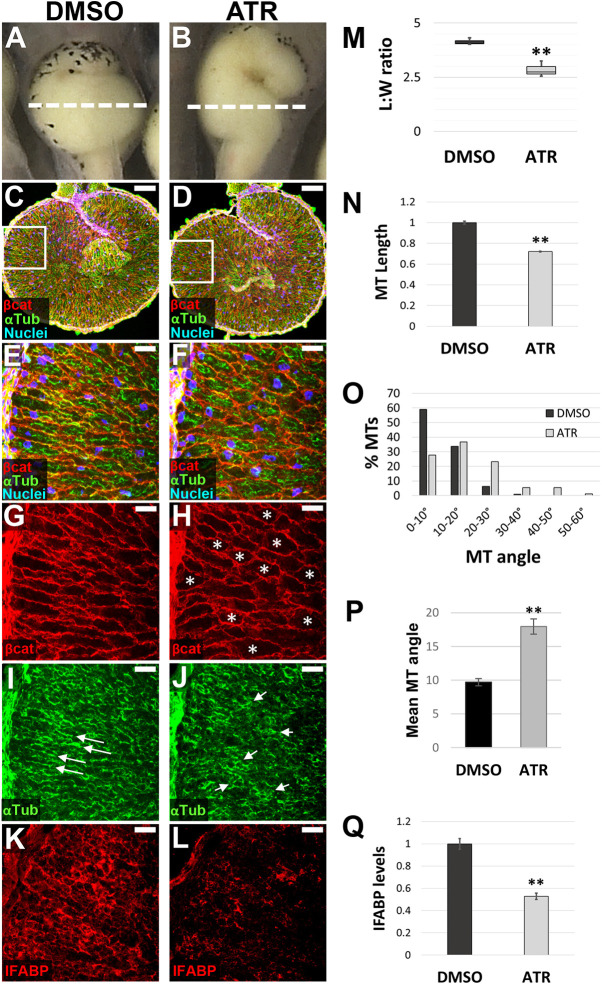
**ATR inhibits endoderm cell properties required for early intestine elongation.** (A-Q) Transverse sections through the intestine of NF 42 control (DMSO; A,C,E,G,I,K) and ATR-exposed (B,D,F,H,J,L) embryos were immunostained for β-catenin (red; C-H) to outline cell membranes, α-tubulin (green; C-F,I,J) to visualize MT bundles, and IFABP (red; K,L) to mark differentiated intestinal epithelial cells. Nuclei (TO-PRO-3) are blue. Dashed lines in A,B indicate the approximate location of DMSO and ATR sections. Boxed regions in C and D are shown at higher magnification in E,G,I and F,H,J, respectively, and approximate the locations of K and L, respectively, in neighboring sections. Note that the control image shown in C is the same as that displayed in Fig. 6A. Cells of ATR-exposed intestines are rounder in shape (G,H, asterisks), as indicated by decreased L:W ratios of individual cells (M), and have short (N), misoriented MT bundles (I,J, arrows; quantified in O,P) and low levels of IFABP (K,L,Q), compared with DMSO controls. Error bars represent s.e.m. **P<0.01 (two-sample *t*-test). Scale bars: 100 µm (C,D); 25 µm (E-L).

By NF 42, the midgut in DMSO-treated embryos had elongated enough to become transversely oriented (Fig. 2A). At the cellular level, the intercalating endoderm appeared radially elongated and polarized (Fig. 2C,E,G), with parallel arrays (bundles) of microtubules (MTs) oriented along the radial (i.e. apicobasal) axes (Fig. 2C,E,I). Simultaneously, expression of the small intestine epithelial marker, intestinal fatty acid binding protein (IFABP; Fabp2), began to reveal nascent differentiation (Fig. 2K; Chalmers and Slack, 2000; Pickett et al., 2017).

In contrast, the midgut in ATR-exposed embryos was visibly shorter than controls (Fig. 2B). Individual endoderm cells in ATR-exposed embryos were rounder, as indicated by their decreased length-to-width (L:W) ratios (Fig. 2D,F,H,M). ATR-exposed endoderm cells also exhibited shorter MT bundles (Fig. 2F,J,N), oriented at aberrant angles (Fig. 2J,O,P), and had decreased expression of IFABP (Fig. 2L,Q). At later stages, analyses of tissue architecture confirmed that the epithelial layer never achieved a normal morphology, instead forming a disorganized, abnormally polarized and sparsely differentiated intestinal lining (Fig. S3). Overall, these data indicate that ATR maintains the endoderm in a more mesenchymal state, inhibiting the polarization, rearrangement and differentiation events required for early intestine elongation and proper epithelial morphogenesis.

### ATR inhibits intestinal cell proliferation

During later stages of intestine morphogenesis (NF43+), cell proliferation becomes an important driver of gut elongation, orchestrated by interkinetic nuclear migration (INM; Marshall and Dixon, 1978; Grosse et al., 2011; Wang et al., 2018, 2019). During INM, dividing endoderm cells delay M phase until the nucleus migrates to the apical surface of the epithelium. After division, the two apically localized daughter cells then become reincorporated into the epithelial layer, extending overall gut length by expanding epithelial surface area (Grosse et al., 2011; Yamada et al., 2013; Wang et al., 2018). Failure to properly execute INM leaves unincorporated daughter cells to die by apoptosis in the gut lumen, compromising the overall rate and extent of intestine elongation (Wang et al., 2018).

**Fig. 3.**
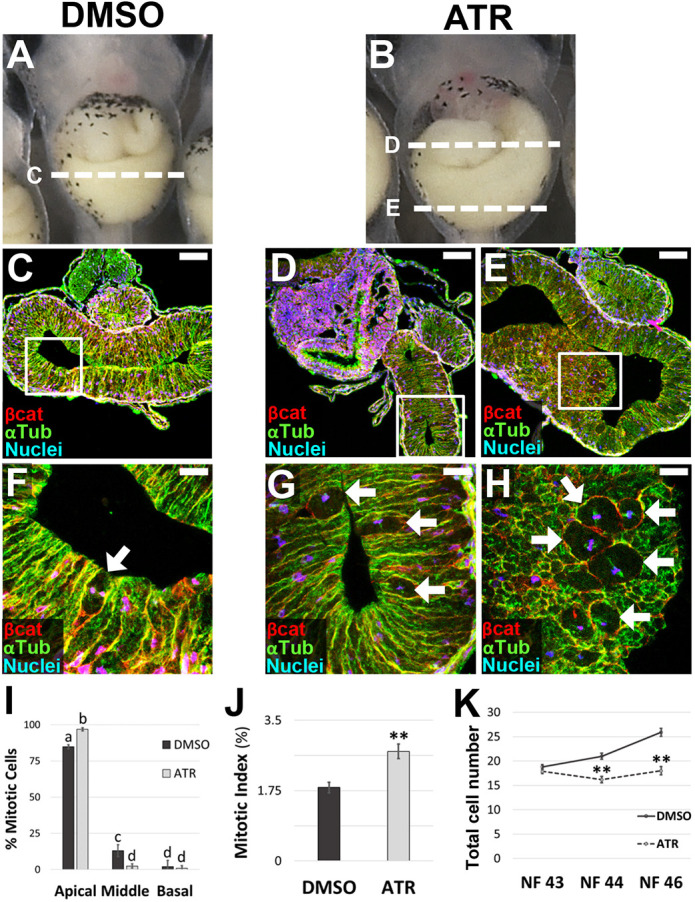
**ATR inhibits epithelial proliferation during late intestinal elongation.** (A-K) Transverse sections through the intestine of NF 44 control (DMSO; A,C,F) and ATR-exposed (B,D,E,G,H) embryos were immunostained for β-catenin (red; C-H) to outline cell membranes and α-tubulin (green; C-H) to visualize MT bundles. Nuclei/chromosomes (TO-PRO-3) are blue. Dashed lines in A,B indicate the approximate location of the sections shown in C-E, with the boxed regions in C-E shown at higher magnification in F-H, respectively. Mitosis occurs almost exclusively near the apical surface of the epithelium in both control and ATR-exposed intestines (arrows, F-I). However, ATR-treated guts have even more apically localized mitotic figures (I), and an increased number of mitotic cells overall, compared with controls (J), including dense clusters of mitoses (E,H). Although total cell number increases over time (NF 43-46) in control intestines, this parameter does not change in ATR-exposed intestines (K). Error bars represent s.e.m. **P<0.01 (I,K, two-sample *t*-test; J, one-way ANOVA with post-hoc Tukey's HSD). Significant differences in I are indicated by distinct lowercase letters (*P*<0.01). Scale bars: 100 µm (C-E); 25 µm (F-H).

To determine whether ATR also affects proliferation-driven gut elongation, we assessed cell division in later stage intestines, when the hairpin loop is forming (NF 43-44). As expected, endoderm cells in DMSO controls (Fig. 3A) underwent mitosis primarily at the apical surface of the gut epithelium (Fig. 3C,F,I). Interestingly, however, in the intestine of ATR-exposed embryos (Fig. 3B), an even greater percentage of mitotic figures were apically localized (Fig. 3D,G,I) such that the overall mitotic index was higher than in controls (Fig. 3J, Fig. S4A-D), with dense clusters of cells in mitosis (Fig. 3E,H). Importantly, however, unlike control intestines, in which total endoderm cell number increased over time (NF 43-46), cell number remained unchanged in ATR-exposed guts (Fig. 3K). In other words, despite the increased mitotic index, the overall cell population did not expand accordingly, suggesting the ATR-exposed mitotic figures are likely arrested in M phase. Moreover, the presence of apoptotic cell debris in the lumens of ATR-exposed embryos at NF 44 indicates that cells had already died and been sloughed into the intestinal lumen (Fig. S4E-I). Overall, these data suggest that ATR inhibits the completion of cell division, leading to mitotic arrest and eventual apoptosis, thereby contributing further to the intestinal elongation and rotation defects in ATR-exposed embryos.

### ATR dysregulates the expression of genes involved in cellular metabolism

**Fig. 4.**
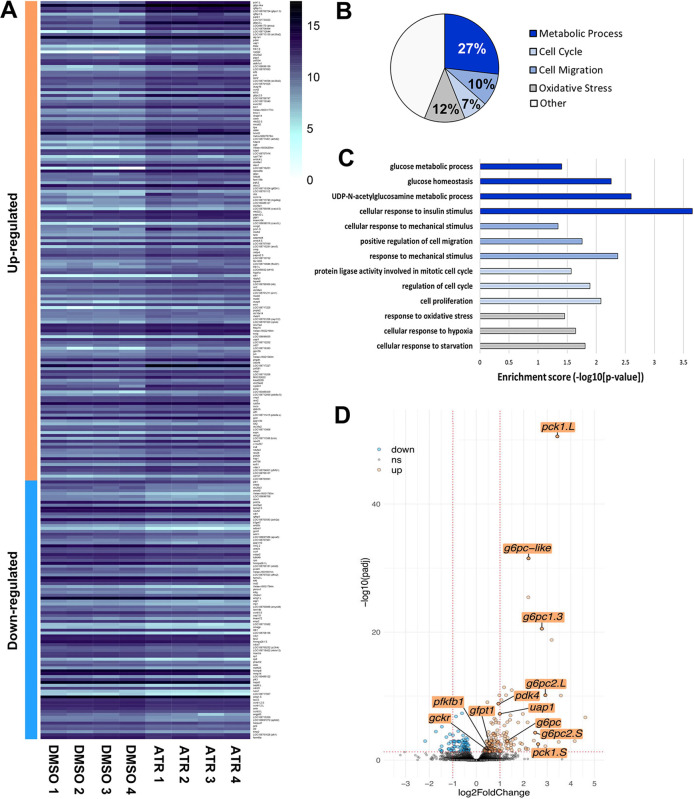
**ATR modulates metabolic gene expression.** (A) Heat map of 254 *X. laevis* intestine transcripts significantly up- or downregulated by 24-h ATR exposure (*P*-adj<0.05; see Table S1). (B) Gene Ontology (GO) analyses indicate that 27% of GO terms were related to metabolic pathways (dark blue), 12% were related to oxidative stress (gray), 10% were involved in cell migration processes (medium blue) and 7% were related to regulation of the cell cycle (light blue). (C) Significance scores reveal enrichment of genes involved in stress responses, cell migration and mechanics, cell cycle regulation and proliferation, and glycolysis-related metabolic pathways. (D) Volcano plot of differentially regulated transcripts (log2-fold-change threshold=1, *P*-value threshold=0.05), highlights the upregulation of relevant glycolysis-related genes. Distinct copies of genes on the two *Xenopus* sub-genomes are designated as .L and .S.

**Fig. 5.**
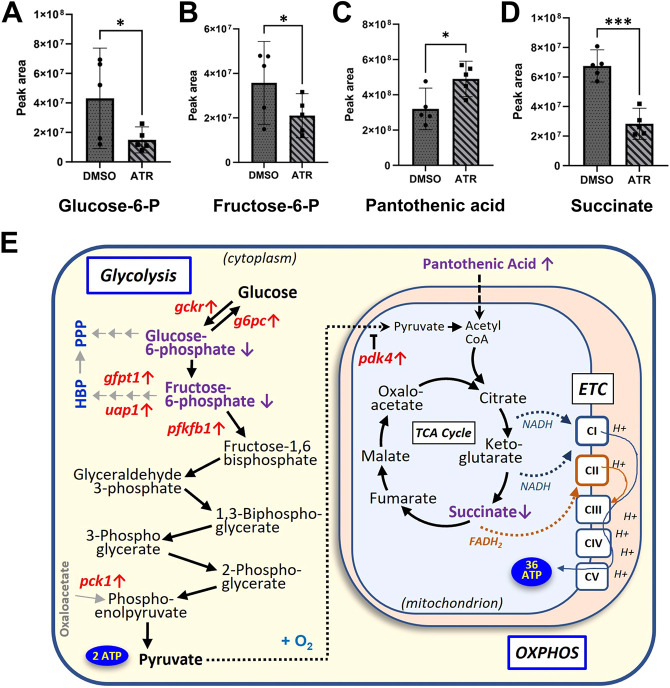
**ATR dysregulates central carbon metabolism in the intestine.** (A-D) Metabolomic analysis of ATR-exposed guts (summarized in Fig. S5) reveals decreased levels of glucose-6-phosphate (Glucose-6-P; A), fructose-6-phosphate (Fructose-6-P; B) and succinate (D), compared with controls, whereas levels of pantothenic acid (C) increased. Error bars in A-D represent 95% confidence intervals. *P≤0.1, ***P<0.01 (Welch’s *t*-test). (E) Diagram of central carbon metabolic pathways showing genes (red) and metabolites (purple) affected by exposure to ATR; up or down arrows next to each gene/metabolite name indicate whether levels were increased or decreased by ATR, respectively. Enzymes encoded by *gckr* and *pfkfb1* regulate key steps of glycolysis; *gfpt1* and *uap1* control the flux of glucose into the hexosamine biosynthesis pathway (HBP); and *g6pc* genes and *pck1* are key regulators of gluconeogenesis. In the presence of oxygen (O_2_), pyruvate crosses the mitochondrial membrane, regulated by the Pdk4 enzyme, which inhibits its conversion to acetyl-CoA and entry into the tricarboxylic acid (TCA) cycle. During a process known as oxidative phosphorylation (OXPHOS), ‘reducing molecules’ generated by the TCA cycle (e.g. NADH, FADH_2_) enable a series of electron transport chain (ETC) complexes (CI-CIV) to build electron (H^+^) potential across the inner mitochondrial membrane. This potential is ultimately used by the last complex, ATP synthase (CV), to generate substantially more ATP (net 36 mols) from the original glucose than glycolysis alone. PPP, pentose phosphate pathway.

To begin to clarify the molecular mechanisms by which ATR causes IM, we performed transcriptome profiling of intestines isolated from embryos exposed to DMSO or ATR for 24 h (i.e. starting at NF 39, harvested at ∼NF 40). Bioinformatic analyses revealed 254 genes that were significantly (*P*-adj<0.05) differentially expressed between control and ATR-exposed guts (Fig. 4A, Table S1). Not surprisingly, Gene Ontology (GO) surveys of the differentially expressed genes revealed enrichment of pathways involved in cell migration and the cell cycle (10% and 7% of total GO terms, respectively), consistent with the observed cellular phenotypes (Fig. 4B,C). However, almost 40% of the enriched pathways were related to cellular metabolism and oxidative stress (27% and 12% of total GO terms, respectively; Fig. 4B,C). Interestingly, genes upregulated by ATR encode enzymes that are important regulators of glycolysis/ gluconeogenesis (e.g. *pck1*, *g6pc* genes, *gckr*, *pfkfb1*) and glycolytic branch pathways, such as hexosamine biosynthesis (e.g. *gfpt1*, *uap1*; Figs 4D and 5E). We also observed upregulation of *pdk4*, a regulatory enzyme that inhibits the pyruvate dehydrogenase complex in the mitochondrial matrix, thereby reducing the conversion of pyruvate to acetyl-CoA that feeds the tricarboxylic acid (TCA) cycle (Figs 4D and 5E). These changes in gene expression suggest that ATR-exposed intestinal cells upregulate glycolytic metabolic pathways and reduce mitochondrial metabolism.

### ATR perturbs central carbon metabolism

To understand further the effects of ATR on central carbon metabolism, we also compared the metabolites present in intestines isolated from DMSO- versus ATR-exposed (24 h) embryos. Metabolomic profiles were acquired by untargeted analysis with inclusion of a targeted mass list for nine central carbon metabolites of interest based on our transcriptomics results (see Materials and Methods). We identified ∼400 annotated features in the *Xenopus* gut, 21 of which exhibited significant (*P*≤0.1) differential abundance in the presence of ATR (Fig. S5).

These profiles revealed the presence of ATR and ATR metabolites (e.g. 2-hydroxyatrazine) in ATR-exposed intestines, confirming uptake of the herbicide by the cells of the developing gut tube (Figs S5, S6A,B). ATR-treated intestines were also found to exhibit increased levels of N-acetyl-L-tyrosine (Figs S5, S6C), a molecule associated with reactive oxygen species (ROS) signaling during mito-hormesis in stressed animals (Matsumura et al., 2020), corroborating the oxidative stress implicated by RNA sequencing.

As suggested by the transcriptome profiles described above, metabolomics analyses also confirmed that ATR exposure specifically impacts several aspects of central carbon metabolism (Fig. 5). For example, within the glycolysis pathway, ATR exposure was found to decrease the levels of two early glycolytic intermediates: glucose-6-phosphate and fructose-6-phosphate (Fig. 5A,B, Fig. S5), consistent with the altered glycolytic/gluconeogenic activity and use of branch pathways (e.g. hexosamine biosynthesis) predicted by transcriptome profiling (Fig. 5E). Moreover, in the presence of ATR, we observed accumulation of a precursor molecule used to generate coenzyme A (CoA) for the TCA cycle (pantothenic acid; Fig. 5C), consistent with decreased consumption of acetyl-CoA predicted by upregulation of the *pdk4* gene (Fig. 5E).

Finally, within the TCA cycle itself, significantly lower levels of succinate were detected in ATR-exposed intestines (Fig. 5D, Fig. S5). Interestingly, the conversion of succinate to fumarate produces reducing molecules (i.e. FADH_2_) that directly fuel mitochondrial ETC Complex II (Fig. 5E). The specific depletion of succinate in the presence of ATR is therefore consistent with ATR-exposed mitochondria bypassing Complex I, the ETC component known to be inhibited by ATR in other contexts (Lim et al., 2009; Karadayian et al., 2023). We also noted a significant decrease in malonic acid, a well-known competitive inhibitor of Complex II, in the presence of ATR (Fig. S5), lending further credence to the idea that ATR-exposed mitochondria are utilizing Complex II to bypass inhibition of Complex I.

### The effects of ATR on endoderm morphogenesis are phenocopied by rotenone

**Fig. 6.**
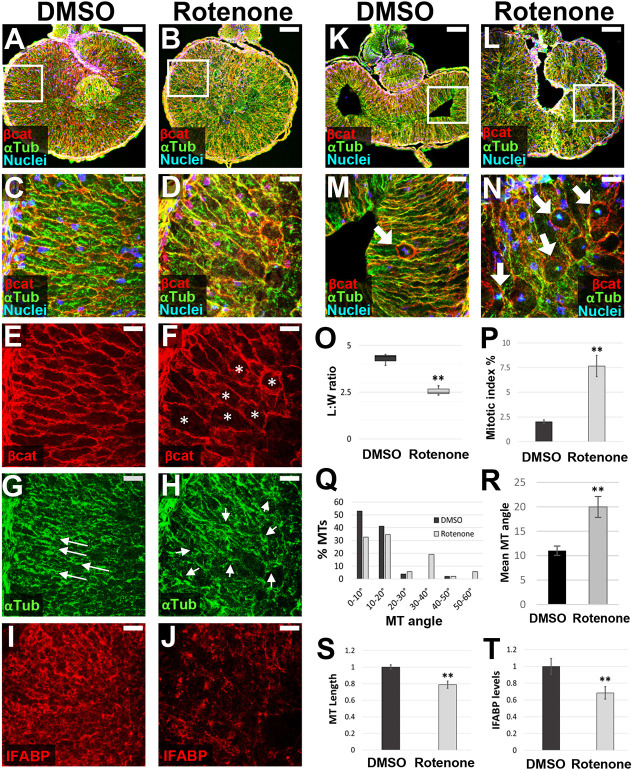
**Inhibiting mitochondrial ETC complex I by rotenone phenocopies ATR.** (A-T) Transverse sections through the intestine of NF 42 (A-J) and NF 44 (K-N) DMSO control (A,C,E,G,I,K,M) and rotenone-exposed (B,D,F,H,J,L,N) embryos were immunostained for β-catenin (red; A-F,K-N) to outline cell membranes and α-tubulin (green; A-D,G,H,K-N) to visualize MT bundles. IFABP (red; I,J) was used as a marker of differentiated intestinal epithelia. Nuclei, blue (TO-PRO-3). Boxed region in A approximates the locations of C,E,G,I in neighboring sections; boxed region in B is shown at higher magnification in D,F,H, and approximates the location of J in a neighboring section. Note that the control image shown in A is reproduced from Fig. 2C. Boxed regions in K,L are shown at higher magnification in M,N, respectively. Compared with DMSO controls, the endoderm cells of rotenone-exposed intestines are rounder in shape (E,F, asterisks), as indicated by decreased L:W ratios of individual cells (O). They also exhibit abnormal polarity, indicated by disoriented and shorter MT bundles (G,H, arrows; quantified in Q-S). In addition, rotenone-exposed cells exhibit lower levels of IFABP (I,J,T), compared with DMSO controls. Finally, rotenone exposure increases the percentage of mitotically arrested cells at NF 44 (M,N, arrows); quantified by pHH3 staining (P). Error bars represent s.e.m. **P<0.01 (two-sample *t*-test). Scale bars: 100 µm (A,B,K,L); 25 µm (C-J,M,N).

The results described above suggest that, as previously observed (Lim et al., 2009; Karadayian et al., 2023), ATR may inhibit electron transport via Complex I of the mitochondrial ETC. To determine whether specifically inhibiting Complex I by another means elicits the same phenotypes as ATR, we exposed embryos to rotenone, a well-characterized Complex I inhibitor known to dysregulate mitochondrial metabolism and compensatorily upregulate glycolysis (Karlsson et al., 2016; Hou et al., 2018; Gonzalez-Hunt et al., 2021). Exposure to rotenone just prior to gut elongation (NF 39) precisely phenocopied the early (NF 42) cellular effects of ATR, causing endoderm cells to be rounder (Fig. 6A-F,O), with disorganized and shorter MT bundles (Fig. 6G,H,Q-S) and decreased levels of IFABP (Fig. 6I,J,T). Moreover, by NF 44, we identified a significantly increased number of mitotic figures in rotenone-exposed intestines (Fig. 6K-N,P). The strong similarity between rotenone- and ATR-induced cellular phenotypes, despite their dissimilar chemical structures, provides further support for the idea that ATR inhibits mitochondrial ETC Complex I in the developing intestine.

### A metabolic shift occurs during intestine morphogenesis

Our results indicate that proper implementation of central carbon metabolism is required to ensure sufficient gut tube elongation and CCW intestine rotation. To further define the normal dynamics of cellular metabolism during gut morphogenesis, we performed bioenergetics assays on endoderm cells isolated from control versus ATR-exposed embryos, using the Agilent Seahorse XFp Analyzer to compare rates of glycolytic versus mitochondrial (OXPHOS) ATP production at successive stages of development.

**Fig. 7.**
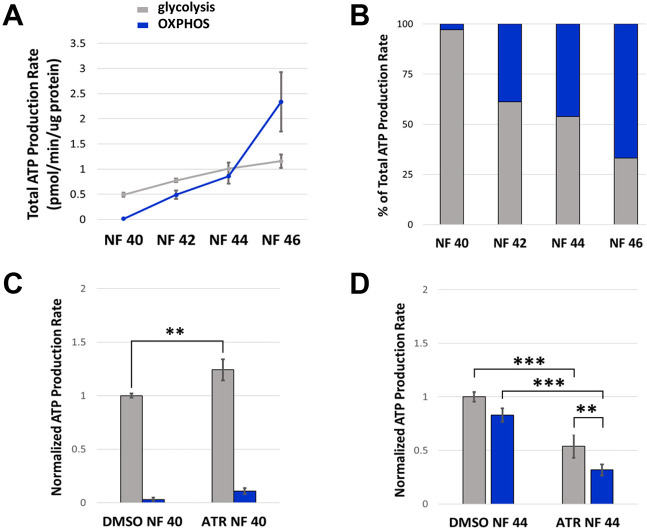
**A metabolic transition is required for intestine morphogenesis.** (A,B) Glycolytic (gray) and mitochondrial (OXPHOS; blue) ATP production rates were measured in control embryos and represented as average levels of ATP production (A) and percent of total ATP production (B) by each pathway. (A) Glycolysis is the predominant pathway for energy production until NF 44, when mitochondria in differentiated epithelia begin to produce nearly equivalent levels of ATP. Subsequently (NF44+), OXPHOS becomes the predominant energy production pathway. (B) At early stages (NF 40), only 3% of ATP comes from mitochondrial processes; however, by NF 46 OXPHOS is the predominant source of ATP (67%). (C) Exposure to ATR increases the glycolytic ATP production rate at early stages (NF 40). (D) At later stages (NF 44), although chronic ATR exposure dampens the rate of energy production by both pathways, glycolysis remains the predominant source of energy in ATR-exposed embryos, producing ATP at almost twice the rate of OXPHOS. All results are from at least three independent experiments with 15-30 embryos each. Error bars represent s.e.m. **P<0.05, ***P<0.01 (one-way ANOVA with post-hoc Tukey's HSD).

This quantification identified a previously unappreciated shift in the predominant metabolic pathway employed by intestinal endoderm during different phases of gut elongation (Fig. 7A,B). For example, at early stages (NF 40), when radial rearrangement is beginning and cell proliferation is low (mitotic index <1%; not shown), glycolysis was the sole source of ATP production. However, mitochondrial ATP production then became evident at NF 42, when differentiation markers are starting to be expressed (see Fig. 2). By NF 44, when the epithelium is largely pseudostratified, intestinal fates are well established and proliferation is starting to propel midgut elongation, the intestine relied equally on both glycolytic and OXPHOS-derived sources of ATP. Finally, by NF 46, when proliferation is the sole driver of continued gut lengthening (mitotic index >4%; not shown), ATP production was powered primarily by OXPHOS. Thus, the metabolic state of the developing intestine undergoes a shift that correlates with the different morphogenetic events driving its elongation.

A parallel analysis of endoderm cells isolated from ATR-exposed intestines revealed that, initially (NF 40), the use of glycolysis for ATP production was upregulated by the presence of ATR (Fig. 7C), consistent with the increased glycolytic activity suggested by transcriptomic and metabolomic profiles. Importantly, however, by NF44, when the gut would normally have substantially upregulated mitochondrial metabolism, ATR-exposed intestine cells remained predominantly glycolytic, continuing to produce almost twice as much ATP by glycolysis as OXPHOS, although total ATP production by both pathways had decreased from the chronic ATR exposure (Fig. 7D). These results suggest that the presence of ATR compels the intestine to rely on glycolysis as its primary metabolic pathway throughout gut morphogenesis, inhibiting the normal shift to OXPHOS that accompanies proper completion of MET and proliferation-driven tissue elongation.

### ATR disrupts redox homeostasis

**Fig. 8.**
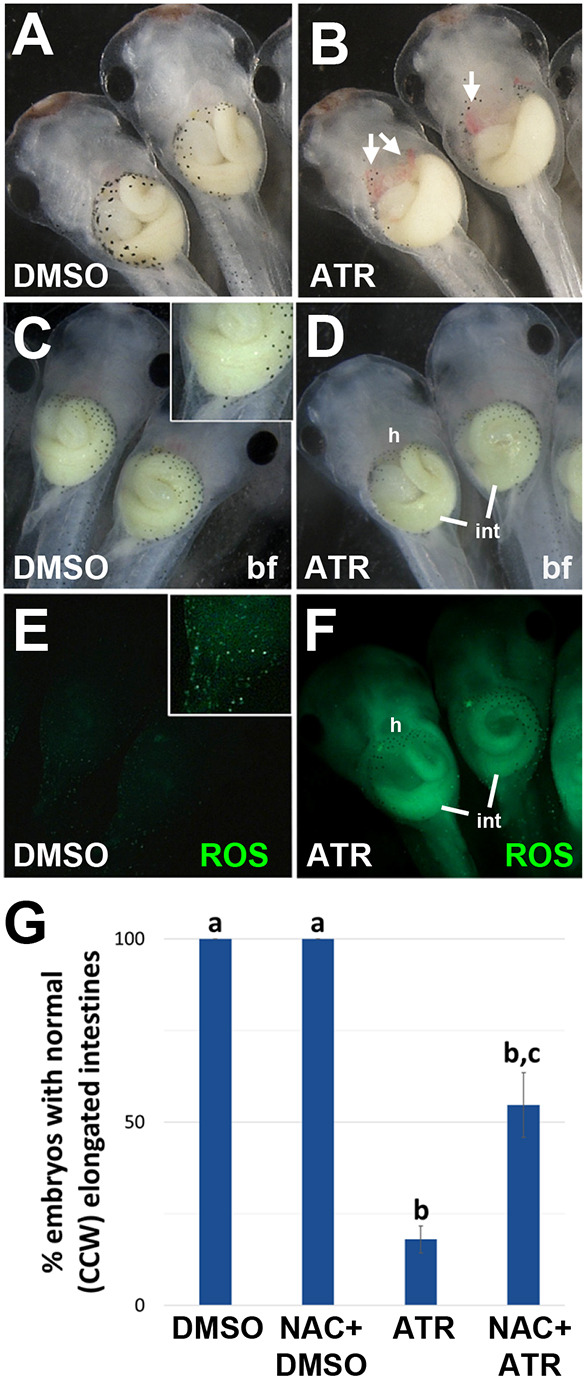
**ATR-induced elongation and rotation defects are rescued by antioxidant pretreatment.** (A,B) Compared with DMSO controls (A), ATR-exposed embryos (B) exhibit visceral hemorrhaging (arrows, B), a sign of oxidative stress. The images in A and B show cropped areas of Fig. S2A and B, respectively. (C-F) Embryos were incubated in DMSO or ATR for 30 min before the addition of a green fluorescent ROS detector (H2DCFDA). After 2 h, embryos were visualized with brightfield (bf; C,D) or fluorescent (E,F) optics. Compared with DMSO controls (E), ATR-exposed embryos (F) exhibit significantly increased (*P*<0.01, one-way ANOVA with post-hoc Tukey's HSD) levels of reactive oxygen species (ROS, green), particularly in the intestine (int) and in the vasculature near the heart (h). Insets in C and E (at higher exposure) reveal individual ROS-positive cells in the normal epidermis. (G) Pretreating embryos with an antioxidant (NAC) does not affect normal gut morphology in DMSO controls, but rescues both the elongation and rotation defects caused by ATR (see also Fig. S7). Antioxidant rescue was quantified by measuring the percentage of guts in each condition with 2+ intestinal coils and normal (CCW) rotation direction; significant differences between treatment groups are indicated by distinct lowercase letters (*P*<0.01, one-way ANOVA with post-hoc Tukey's HSD). All results are from at least three independent experiments with 8-15 embryos each.

Chronic inhibition of electron transport reactions in ATR-exposed embryos is likely to overwhelm endogenous antioxidant defenses, leading to redox imbalance (Pisoschi et al., 2021). Indeed, similar to previous reports (Lenkowski et al., 2008), we note that ATR-exposed embryos showed physiological signs of oxidative stress, including visceral hemorrhaging, especially around the heart (He et al., 2020; Fig. 8A,B; Kümin et al., 2007). This was accompanied by a substantial increase (*P*<0.01) in levels of ROS near the heart and in the developing intestine (Fig. 8C-F).

To determine the extent to which aberrant ROS-mediated signaling might influence the IM phenotype, we pretreated embryos with an exogenous antioxidant (N-acetyl-cysteine, NAC) prior to DMSO or ATR exposure. NAC supplementation had no effect on gut morphogenesis in DMSO controls (Fig. 8G, Fig. S7A,B) but, remarkably, restored normal gut morphology – both length and CCW rotation – in a significant proportion of ATR-exposed embryos (Fig. 8G, Fig. S7C,D). These results show that the IM phenotype elicited by ATR is at least partly attributable to the redox imbalance caused by disrupting the metabolic state of the developing gut tube.

## DISCUSSION

It is well established that a change in metabolic status can impact ‘non-metabolic’ cell properties, such as stemness and regeneration (Folmes et al., 2012; Shyh-Chang et al., 2013; Ryall et al., 2015), but the role of metabolic dynamics in regulating the three-dimensional morphogenesis of individual developing organs is just beginning to be explored. In this study, we show that exposure to a photosynthesis-blocking herbicide, ATR, disrupts central carbon metabolism in the developing *Xenopus* gut by inhibiting mitochondrial electron transport reactions, thus decreasing OXPHOS-driven ATP production, prolonging the early glycolytic state, and disrupting redox homeostasis. This abnormal metabolic state disrupts several cellular morphogenetic events in the gut epithelium that are required to impel normal tissue elongation. The resultant decreased length of the gut tube consequently prevents the crucial hairpin loop of the intestine from achieving its normal anatomical position, compelling the intestine to coil in a reversed orientation. Our results thus provide new insight into the timing and nature of the developmental events that influence intestinal rotation while illuminating the potential etiology of a common birth anomaly.

### Metabolic states are dynamic during intestine morphogenesis

**Fig. 9.**
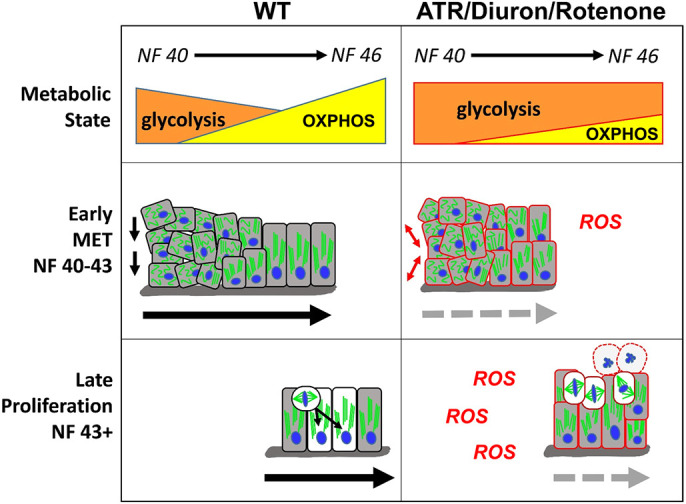
**Model for how cellular metabolism affects gut elongation.** Left: At early stages of gut morphogenesis (NF 40-43), cell rearrangements in wild-type (WT) embryos are promoted by a primarily glycolytic metabolism. Gradually, as a single-layer epithelium is established, increasing OXPHOS supports the completion of MET and intestine cell differentiation. At late stages (NF 43+), the more energy-efficient mitochondrial (OXPHOS) metabolism is also required to support increased rates of proliferation and INM. Right: Inhibition of mitochondrial ETC function by metabolic perturbagens (ATR/Diuron/rotenone) decreases OXPHOS, elevates ROS and prolongs glycolytic activity, thereby preventing the completion of MET and retaining a disorganized epithelium of undifferentiated cells. Continued inhibition of OXPHOS and consequent oxidative stress at later stages retains the primarily glycolytic metabolic state, leading to mitotic arrest and eventual apoptosis (round cells with dashed outlines). Combined, the perturbation of both early (MET) and late (proliferation) elongation processes results in short intestines that fail to achieve the length necessary to undergo proper rotation.

As the *Xenopus* intestine develops, it undergoes a metabolic transition from a predominantly glycolytic state to increased reliance on mitochondrial OXPHOS, correlating with the key cellular morphogenetic events required for gut elongation and rotation (Fig. 9, left). For example, early in gut morphogenesis (NF 40), when endoderm cells exhibit largely mesenchymal properties, glycolysis alone fuels the rearrangements that thin the nascent epithelium. As the intestine starts to lengthen (NF 42-43), a gradual increase in OXPHOS activity coincides with progressive differentiation of the endoderm into a polarized intestinal epithelium (i.e. MET). Eventually, when MET is complete and proliferation drives gut elongation (NF 43-46), mitochondrial metabolism becomes the predominant source of ATP.

Exposure to ATR (and other mitochondrial ETC inhibitors, e.g. Diuron, rotenone) blocks this metabolic transition, extending the early glycolytic state and elevating ROS levels (Fig. 9, right). This aberrant metabolic condition maintains mesenchymal character in the endoderm, preventing the proper completion of early tissue-elongating cell rearrangements and MET/differentiation. At later stages, the less efficient (i.e. glycolysis-dominant, high ROS) metabolism also perturbs cell division, resulting in mitotic arrest and apoptosis rather than increased gut length. Thus, inhibiting the normal transition from largely glycolytic to mainly mitochondrial metabolism specifically hinders the key cellular morphogenetic events required to shape large-scale gut morphology, leading to intestinal shortening and consequent malrotation.

Our observations are consistent with an emerging picture of the role of metabolism in epithelial-mesenchymal plasticity and cell migration during development. For example, glycolysis is required for the initial delamination and migration (i.e. epithelial-to mesenchymal transition, EMT) of mesenchymal neural crest cells from the neural tube, whereas the onset of OXPHOS is associated with their eventual differentiation at their final destination (i.e, MET; Bhattacharya et al., 2020, 2021). Likewise, it has been shown that the rearrangements of the posterior pre-somitic mesoderm that promote elongation of the amniote body axis are primarily fueled by glycolysis, whereas anteriorly the differentiation of this same tissue into somites correlates with a gradual transition to OXPHOS (Bulusu et al., 2017; Oginuma et al., 2017). Interestingly, the signal transduction pathways associated with such metabolic transitions, e.g. FGF and Wnt signaling (Oginuma et al., 2017, 2020), are also known to regulate multiple aspects of epithelial morphogenesis and differentiation along the developing gut tube (Danopoulos et al., 2017; Lv et al., 2019; Kostouros et al., 2020).

Exactly how changes in central carbon metabolism/metabolites might affect cell properties and behaviors is still an emerging area, but intriguing links between metabolism and morphogenesis suggest possible mechanisms. For example, glycolytic enzymes can bind cytoskeletal components, allowing for localized ATP production at the site of cytoskeletal remodeling, thus enabling migratory cell behaviors to be directly promoted by levels of glycolytic activity (Norris et al., 2013; Marelli-Berg and Jangani, 2018). Glycolytic pathways can also influence post-translational protein modifications, such as glycosylation, or generate changes in intracellular pH that affect signal transduction (Oginuma et al., 2020; Sinclair et al., 2021). Cells also coordinate cell division checkpoints with their metabolic state. For instance, genes required for cell cycle progression and energy homeostasis are often transcribed together (Kalucka et al., 2015; Salazar-Roa and Malumbres, 2017), and the presence of a functional ETC is a prerequisite for cell-cycle progression (Wang et al., 2014). Given that temporally and spatially regulated cell rearrangement and/or cell division events underlie the proper formation of numerous embryonic tissues, developmental regulation of central carbon metabolism may be a general mechanism of shaping the 3D form of many developing organs. Consequently, abnormal metabolic status and/or redox imbalance during organogenesis may contribute to the etiology of a wide variety of structural birth anomalies beyond the gut.

### ATR disrupts mitochondrial metabolism and redox balance during gut morphogenesis

Our observations suggest that ATR blocks the normal transition to OXPHOS-driven metabolism during gut morphogenesis by inhibiting mitochondrial electron transport via Complex I. For example, we showed that identical cellular phenotypes are induced by exposure to rotenone, a known Complex I inhibitor. In addition, the reduced succinate levels and decreased mitochondrial ATP production rate suggest that ATR-exposed guts do not effectively utilize ETC Complex I, instead resorting to (suboptimal) OXPHOS via Complex II. Finally, the increased ROS levels in ATR-exposed embryos are also consistent with inhibition of electron transport reactions. Importantly, we found that both elongation and rotation defects can be partially rescued by antioxidant supplementation, confirming that at least part of the mechanism by which ATR induces IM is via redox imbalance. In both normal and pathogenic contexts, ROS have been shown to modulate multiple redox-sensitive pathways influencing cell migration (cytoskeletal reorganization and function; Wilson and González-Billault, 2015), EMT/MET plasticity (Chatterjee and Chatterjee, 2020), cell fate/differentiation (Covarrubias et al., 2008) and cell cycle phase transitions (Burhans and Heintz, 2009); thus, elevated ROS signaling alone could concomitantly perturb all the gut elongation events compromised by ATR.

Consistent with decreased mitochondrial functioning, we also find that ATR compensatorily upregulates transcription of *pdk4*, an enzyme that inhibits the utilization of pyruvate (the end product of glycolysis) by the mitochondrion, as well as multiple enzymes regulating glycolytic pathways. Concomitant decreases in levels of key glycolytic metabolites (glucose-6-phosphate and fructose-6-phosphate) suggest that carbon molecules not able to be utilized by mitochondria may be redirected via gluconeogenesis enzymes (G6pc or Pck1), or used to fuel post-translational protein modifications through the hexosamine biosynthetic pathway (via Gfpt1/Uap1), perhaps maintaining stemness (Akella et al., 2019), which could also alter endoderm cell properties and behavior.

Further investigation is needed to determine how ATR may affect mammalian gut morphogenesis, but we note that gestational exposure to ATR has been epidemiologically associated with several human congenital anomalies (Winchester et al., 2009; Waller et al., 2010; Agopian et al., 2013; Almberg et al., 2018; Politis, 2018), including cases of gastroschisis, which, coincidentally, often exhibit IM (Forrester and Merz, 2008; Hunter and Stevenson, 2008; Choi et al., 2018; Ishii et al., 2020). Our results suggest that abnormal mitochondrial metabolism, caused by, for example, environmental perturbagens (such as ATR), fetal gene variants, and/or maternal metabolic disease, has the potential to derail conserved cellular events during gut morphogenesis and may therefore be a previously unappreciated cause of abnormal intestinal anatomy. Our findings also raise the possibility that milder metabolic insults may elicit more subtle alterations of endoderm cell morphology and behavior that do not affect intestine rotation per se, but may nonetheless compromise intestinal length, epithelial architecture and/or barrier function, as relevant to a broad range of digestive diseases (e.g. celiac or inflammatory bowel disease). Finally, our study raises the intriguing possibility that, as noted for other birth anomalies caused by redox-active teratogens (Dennery, 2007; Pace et al., 2018), adequate antioxidant consumption in the periconceptional diet could have a protective effect against such insults.

### Gut elongation is integral to rotation

Human IM phenotypes are often attributed to defects in embryonic left–right asymmetry (Ticho et al., 2000; Shiraishi and Ichikawa, 2012; Kothari, 2014). In amniotes, the dorsal mesentery (DM), a thick stalk of tissue that suspends the gut tube from the dorsal body wall, has been shown to undergo a left–right asymmetric deformation that tilts the attached gut tube to the left; this early asymmetry is then thought to bias future CCW rotation (Davis et al., 2008). Interestingly, however, the DM is a comparatively insignificant tissue layer in frogs, consisting of literally only a few cells sitting at the top of a massive gut tube that is physically constrained from tilting. Yet the frog intestine still rotates CCW, suggesting that vertebrate intestine morphology may be shaped by multiple mechanisms, including later-stage events such as gut elongation. Indeed, we found that ATR induces IM at late stages, well after the establishment of the left–right body axis and the successful morphogenesis of normal left–right asymmetry in the heart, stomach and liver (Grzymkowski et al., 2020).

Importantly, the window of susceptibility during which ATR elicits IM phenotypes coincides precisely with the period of gut tube elongation. The fact that antioxidant pretreatment (or ATR washout) simultaneously rescues length and CCW coiling at these late stages suggests that, remarkably, simply restoring normal elongation may be sufficient to restore proper rotation. Moreover, other studies have noted that experiments designed to specifically perturb gut length (independent of left–right asymmetry) often cause coincidental malrotations in *Xenopus* (Lipscomb et al., 2006; Reed et al., 2009; Pickett et al., 2017; Dush and Nascone-Yoder, 2019) and mice (Yamada et al., 2010).

In humans, intestine rotation also coincides with the period of most rapid gut elongation, and it has been suggested that, rather than a failure to execute an *en masse* ∼270° rotation, certain IM phenotypes may instead result from incomplete lengthening of different regions of intestine, which can result in abnormal positioning of particular intestine loops (Soffers et al., 2015; Grzymkowski et al., 2020). Consistent with this idea, individuals with IM often present with additional developmental anomalies (congenital short gut, stenoses or atresias) that locally compromise intestine length (Hamilton et al., 1969; Kern et al., 1990; Erez et al., 2001; Chu et al., 2004; Hasosah et al., 2008; Martin and Shaw-Smith, 2010; Shehata et al., 2011; Adams and Stanton, 2014; Negri et al., 2020). Thus, there appears to be an inextricable relationship between the process of gut lengthening and the process of gut rotation across species.

Our discovery of the ability of a metabolic perturbagen (ATR) to induce malrotation at late stages recontextualizes IM as a possible defect in gut elongation, potentially independent from earlier defects in left–right asymmetry. This provides a novel alternative explanation for the many cases of human IM that occur sporadically, and/or are clinically associated with inadequate gut elongation. Given that multiple molecular pathways and morphogenetic processes impact gut lengthening (Zhang et al., 2001; Yamada et al., 2010; Grosse et al., 2011; Wang et al., 2018; Dush and Nascone-Yoder, 2019), a wide array of developmental deficiencies, including but not limited to metabolic insults, could contribute to the high prevalence and variability of intestinal malrotation phenotypes.

## MATERIALS AND METHODS

### Embryo culture and chemical treatment

*Xenopus laevis* embryos were obtained by *in vitro* fertilization, de-jellied using 2% cysteine pH 7.9, and raised in 0.1× Marc's Modified Ringers (MMR) at 14-23°C. Embryos were staged according to Nieuwkoop and Faber (NF; Faber, 1994; Zahn et al., 2022).

### Chemical exposures

NF 39 (early tadpole stage) embryos were exposed to a range of concentrations of atrazine (ATR; 15-35 mg/l), Diuron (35 mg/l), rotenone (155-200 nM) or an equivalent volume of solvent (DMSO or ethanol) and incubated at 14-16°C until NF 46 (coiled intestine).

To detect ROS levels, embryos (NF 42-46) were incubated in DMSO or 35 mg/l ATR for 30 min before the addition of 15 µM H_2_DCFDA (Thermo Fisher Scientific) for 2 h at 23°C. Fluorescence was documented using a Zeiss Lumar stereoscope and quantified in Fiji by measuring the mean fluorescent intensity of H_2_DCFDA of the embryo minus mean fluorescent intensity of the background.

To attempt to rescue redox imbalance, NF 39 embryos were incubated in NAC (1.2 mM) for 2 h before being exposed to ATR (35 mg/l) or DMSO as described above.

### Cryosectioning and immunohistochemistry

Embryos at multiple developmental stages (NF 40-44) were fixed in 4% paraformaldehyde (in PBS) for 45 min at room temperature, followed by eight washes in Dent's fixative (80% methanol/20% DMSO). Fixed embryos were stored in Dent's at −20°C until infiltration. Embryos were then rinsed and incubated in Tris-NaCl (100 mM Tris pH 7.3, 100 mM NaCl) for 30 min before being incubated overnight in a solution of 15% sucrose/25% cold water fish gelatin. Embryos were then embedded in Tissue-Plus™ O.C.T. Compound (Thermo Fisher Scientific) and 10-μm-thick sections were obtained using a Leica CM1860 cryostat and thaw-mounted on glass slides before being dried overnight.

Sections were post-fixed in acetone for 1 min, incubated in a 20% SDS solution for 3 min, rinsed three times in 1× PBS, and blocked for 30 min in blocking buffer (0.1 M Tris pH 7.4, 0.15 M NaCl, 0.05% Tween 20, 5% horse serum, 0.5% casein, Hammarsten grade). The following primary antibodies were added at the following dilutions and allowed to incubate overnight at 4°C: mouse anti-E-cadherin (clone 5D3, Developmental Studies Hybridoma Bank; 1:200), mouse anti-integrin (clone 8C8, Developmental Studies Hybridoma Bank; 1:1000), anti-β-catenin (71-2700, Thermo Fisher Scientific; 1:200), anti-α-tubulin (T9026, Sigma-Aldrich; 1:1000), rabbit anti-IFABP (gift from Yun-Bo Shi, NIH, Bethesda, MD, USA; 1:1000), rabbit anti-cleaved caspase-3 (9661T, Cell Signaling Technology; 1:300), anti-pH3 (06-570, Millipore-Sigma; 1:500). Slides were then washed twice in 1× PBS and incubated for 1.5 h in blocking buffer containing secondary antibodies: goat anti-mouse/rabbit conjugated with Alexa Fluor 488/555 (A11029 and A21428, Thermo Fisher Scientific; 1:2000). Slides were subsequently washed twice in 1× PBS and incubated with a 1:1000 dilution of TO-PRO-3 (T3605, Thermo Fisher Scientific) in 1× PBS for 30 min to visualize nuclei. Finally, they were mounted with Prolong Gold (P36930, Thermo Fisher Scientific) and a coverslip before sealing with nail polish. Mounted slides were stored at −80°C until visualization (Leica TCS SPE confocal microscope).

### RNA sequencing and analysis

NF 39 embryos were exposed to 35 mg/l ATR or DMSO for 24 h. Ten intestines from each treatment group (four biological replicates) were dissected from anesthetized embryos with sharpened forceps and collected into microcentrifuge tubes containing TRIzol™ Reagent (Invitrogen). RNA extraction was performed according to the manufacturer's instructions, followed by lithium chloride precipitation. Quality and quantity of RNA yield was confirmed on a Thermo Fisher Scientific Nanodrop 1000 spectrophotometer and an Agilent 2100 Bioanalyzer. RNA sequencing was performed by North Carolina State University's Genomic Sciences Laboratory (Raleigh, NC, USA) with an Illumina poly-A-enriched directional RNA library. An Illumina NovaSeq 6000 was used for sequencing (SP 150 bp PE flow cell).

RNA-sequencing data analysis was performed in consultation with the Bioinformatics Core at the NCSU Center for Human Health and the Environment. An average of ∼62.5 million paired-end raw sequencing reads were generated for each replicate. The quality of sequence data was assessed using the fastQC application, and ten (poor-quality) bases were trimmed from the 5′ end. The remaining good quality reads were aligned to the *Xenopus laevis* reference genome (ZENLA 9.2) database using the STAR aligner (Dobin et al., 2013). Per-gene counts of uniquely mapped reads for each replicate were calculated using the htseq-count script from the HTSeq python package (Anders et al., 2015). The count matrix was imported to the R statistical computing environment for further analysis. Initially, genes that had no count in most replicate samples were discarded. To assess for outliers, a regularized log transformation function (DESeq2) that transforms the count data into the log2 scale and minimizes differences between samples for rows with small counts, was then applied, and a principal component plot was constructed using the top 500 variable genes (Fig. S8). The count data were then normalized for sequencing depth and distortion, and dispersion was estimated using the DESeq2 Bioconductor package in the R statistical computing environment (Love et al., 2014). A linear model was fitted using the treatment levels, and differentially expressed genes were identified after applying multiple testing corrections using the Benjamini–Hochberg procedure (Benjamini and Hochberg, 1995). The final significant genes were generated using *P*-adj<0.05.

The R packages ‘ggplot2′ and ‘pheatmap’ were used to create figures (Wickham, 2016; https://cran.r-project.org/web/packages/pheatmap/index.html). Heatmap only includes significant genes (*P-*adj≤0.05).

### Metabolomics

NF 39 embryos were exposed to 35 mg/l ATR or DMSO control for 24 h. Thirty intestines (15 mg±0.3 mg) were collected from each treatment group (five biological replicates), flash-frozen and stored at −80°C. Metabolite extraction, data collection and analysis were performed by NC State University's Molecular Education, Technology and Research Innovation Center (METRIC). Polar metabolites were extracted in 80% methanol, chilled at −80°C and contained internal standards [Metabolomics QReSS™ Kit and D-glucose (U-13C6, 99%), Cambridge Isotope Laboratories, Tewksbury, MA, USA] for quality control. Samples were then homogenized using a Geno/Grinder with a 2.8-mm stainless steel grinding ball (SPEX) and incubated at 80°C for 1 h. After a 10-min centrifugation at 20,000 ***g*** at 4°C, the supernatant was removed and the pellet was re-extracted with cold 80% methanol, incubated at −80°C for 30 min and the supernatants were combined. Pooled QC samples were made by combining 35 μl of each sample into one microcentrifuge tube. For each sample, 350 μl of extract was dried in a vacuum concentrator (Savant SpeedVac SPD140DDA, Fisher Scientific). Polar metabolites were reconstituted in 25 μl of Optima grade water followed by 75 μl of Optima grade acetonitrile. Extracted samples were analyzed using hydrophilic interaction liquid chromatography-mass spectrometry (HILIC-MS) for polar metabolites.

### Liquid chromatography–mass spectrometry conditions for metabolomics

Chromatographic separation was achieved using an Atlantis Premier BEH Z-HILIC VanGuard FIT column (1.7 µm, 2.1 mm×100 mm) and a similar chromatographic method to that described previously (Smith et al., 2021). Mobile phase A consisted of 15 mM ammonium bicarbonate in water (pH 9.0) and mobile phase B was 15 mM ammonium bicarbonate in 9:1 (v/v) acetonitrile:water, with the following elution profile: 0 min 90% B, 15 min 65% B, 16 min 65% B, 16.5-22 min 90% B. The flow rate was 0-16.5 min 0.5 ml/min, 16.5-21.5 min 1 ml/min, 21.51-22 min 0.5 ml/min. The injection volume was 2 μl and 10 μl for positive and negative ionization modes, respectively. An Orbitrap ID-X Tribrid Mass Spectrometer (Thermo Fisher Scientific) with electrospray ionization, coupled to a Vanquish Horizon UHPLC system was used for data acquisition. Ionization source conditions were: sheath gas flow 50 arbitrary units (Arb), auxiliary gas flow 10 Arb, sweep gas flow 1 Arb, ion transfer tube temperature 300°C and vaporizer temperature 250°C. Spray voltages were 3.5 kV and 2.5 kV for positive and negative polarity, respectively. Data were acquired with a scan range of m/z 70-700, and MS1 resolving power set to 120,000 FWHM at m/z 200, with an automatic gain control (AGC) target of 1e5 and a maximum injection time of 50 ms. The RF lens was 60%. The intensity threshold was set to 1e4. For data-dependent MS2 (ddMS2) acquisition, the resolving power was set to 30,000 with standard AGC target and a maximum injection time of 54 ms. A targeted mass list was included for MS2 prioritization of several metabolites in pathways implicated by initial transcriptomics studies; glucose, pyruvate, citrate, fumarate, 2-hydroxyglutarate, 2-oxoglutarate, malate, lactate and G6P were added to this list to ensure fragmentation of these analytes if present in MS1 full scans. The MS2 isolation window was set to 1.6 m/z and normalized collision energies of 20%, 35% and 60% were used as the stepped collision energy. The dynamic exclusion was set to 2.5 s. AcquireX Deep Scan was conducted on pooled QC samples in negative ionization mode to increase the metabolite coverage by obtaining MS2 fragmentation information on less abundant metabolites in the sample. Data files (.raw) can be retrieved from PanoramaWeb (https://panoramaweb.org/__r11638/project-begin.view).

### Metabolomics data analysis

Compound Discoverer 3.3 (Thermo Fisher Scientific) was used for untargeted metabolomics data analysis, including data visualization, statistics for comparative analyses and compound identification by mzCloud (an online mass spectral database) and mzVault (an in-house spectral library built from authentic standards); identification criteria were MS2 fragmentation match, mass accuracy below 2 ppm and isotopic pattern match. Univariate analysis was performed in Compound Discoverer to determine metabolites that were dysregulated between DMSO and ATR based on Log_2_ fold change ≤−0.8 or ≥0.8 and *P*≤0.1. For reporting relative quantification, Skyline Daily (Adams et al., 2020) was used for peak area integrations prior to significance testing. Statistical significance was determined by Welch's *t*-test, performed using GraphPad Prism version 9.5.1 for Windows (GraphPad Software).

### Seahorse XF real-time ATP rate assay

NF 39 embryos were exposed to DMSO or 35 mg/l ATR until NF 40, NF 42, NF 44 or NF 46. Intestines from 15-20 embryos per treatment group were then microdissected as described above and combined in a microcentrifuge tube. Samples were dissociated in 100 μl of Accumax for 20 min. When the intestines were fully dissociated, samples were centrifuged at 300 rpm (100 ***g***) for 45 s, resuspended in 450 μl Seahorse XF complete DMEM media, pH 7.4 (10 mM glucose, 1 mM pyruvate, 2 mM L-glutamine), and 150 μl of each sample was added to an individual well of an 8-well Seahorse microplate coated with poly-D-lysine (three technical replicates per sample). The microplate was gently spun to adhere the cells to the bottom of the well. The total volume of each well was then brought up to 180 μl with DMEM, and the microplate was incubated at 28°C for 1 h. Following incubation, the Seahorse XF Real-Time ATP Rate assay was run on a Seahorse XFp analyzer according to the manufacturer's instructions. After the assay run, the cells from each well were collected and protein was extracted in RIPA Buffer and quantified using the Pierce™ BCA Protein Assay kit (Thermo Fisher Scientific). Raw ATP rate values were normalized for differences in protein amount between individual wells.

### Morphometric measurements and statistical analysis

Intestine length was quantified by counting the number of intestinal loops visible in NF 46 whole embryos, a method validated by directly measuring the lengths of isolated intestines (Fiji). The average length to width (L:W) ratio of individual gut endoderm cells was calculated from measurements (Fiji) of the maximum lengths and widths of representative cells (*n*=5) in NF 42 sections from three different experiments (*n*=15 per condition). Average MT bundle length was inferred from measurements of the maximum length of visible MT bundles (*n*=20-40) in NF 42 intestine sections from three different experiments (*n*=50-120 per condition). All average values were normalized to controls. Differences in MT polarity were quantified by measuring the angle of deviation of individual MT bundles (*n*=20-40) in relation to the apicobasal axis (0°) of NF 42 intestine sections from three different experiments (*n*=50-120 per condition). IFABP levels were quantified in section by measuring the mean immunohistochemical signal intensity of the tissue minus the mean signal intensity of the background (Fiji); average levels were normalized to controls. To assess proliferation, the numbers of phospho-Histone H3 (pHH3)-positive cells were counted in NF 44 intestine sections (Fiji) from three different experiments (similar results were obtained by counting numbers of cells in mitosis based on chromatin morphology and MT spindle architecture). Total cell population was calculated by counting the numbers of individual cells per square area (*n*=3) within transverse sections (*n*=3-7) through the posterior duodenum and midgut of at least three embryos per stage. Statistical significance was determined by a two-sample *t*-test or one-way ANOVA with post-hoc Tukey HSD.

## Supplementary Material

10.1242/develop.202020_sup1 Supplementary information

Table S1. List of genes differentially expressed in guts exposed to ATR

## The people behind the papers

This article has an associated ‘The people behind the papers’ interview with some of the authors.
