# Supplementary material for: Developmental regulation of cellular metabolism is required for intestinal elongation and rotation
Source: Development. 2024 Feb 19;151(4):dev202020. doi: 10.1242/dev.202020 (PMC10911142; doi:10.1242/dev.202020)
Supplement: Supplementary information [file develop-151-202020-s1.pdf]

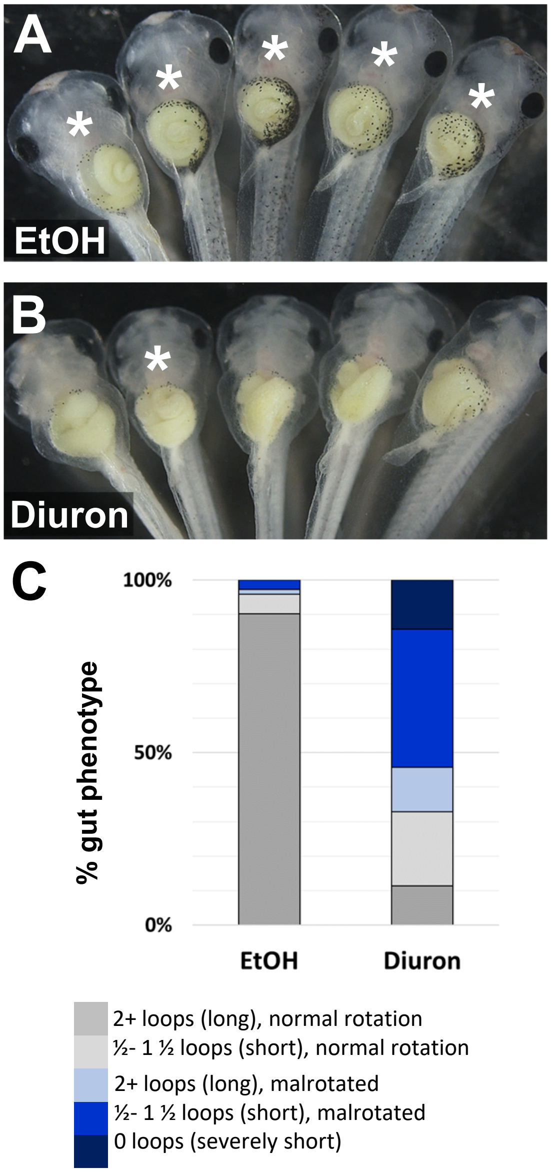

Fig. S1. Exposure to Diuron causes intestinal shortening and malrotation.

(A) EtOH controls develop elongated intestines that are normally rotated (indicated by *), while Diuron-exposed embryos develop intestines that are predominantly short and/or malrotated (B), identical to the phenotypes seen after ATR exposure. Similar to ATR, Diuron elicits varying degrees of phenotypic severity, with short and malrotated being the most prominent (C; royal blue portion of bar). All results shown are from at least three independent experiments with 15-20 embryos each.

Development • Supplementary information

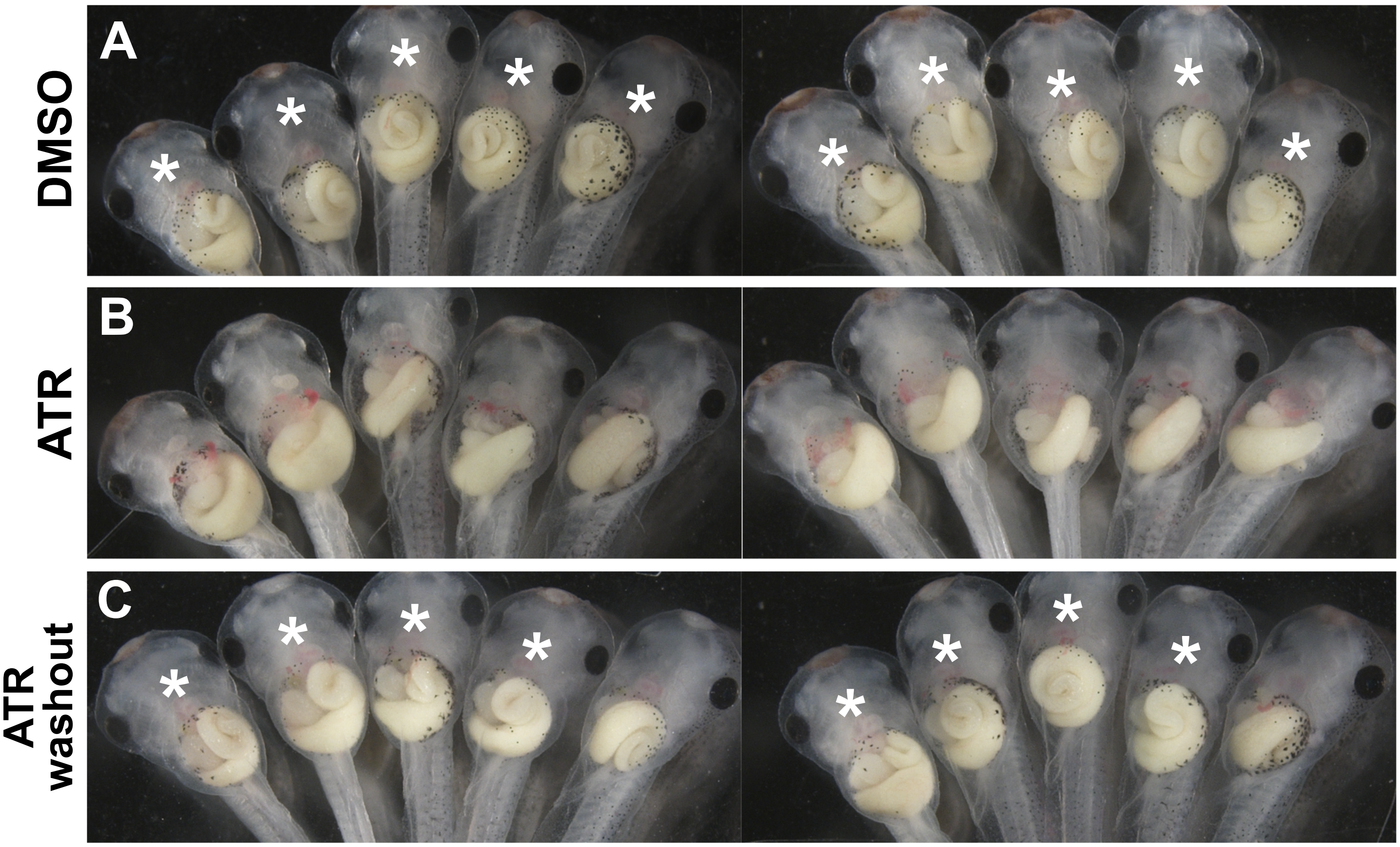

Fig. S2. Removing ATR after initial exposure rescues intestinal shortening and malrotation. DMSO control embryos develop elongated intestines that are normally rotated (indicated by *; A), while embryos exposed to chronic ATR exposure develop intestines that are both short and malrotated (B). C) Most embryos in which ATR was washed out prior to formation of the hairpin loop (by NF 42-43) exhibit greater intestine length and normal CCW rotation. Results were replicated in at least three independent experiments with 8-10 embryos each. Cropped areas from A and B are shown in Fig. 8A and B, respectively.

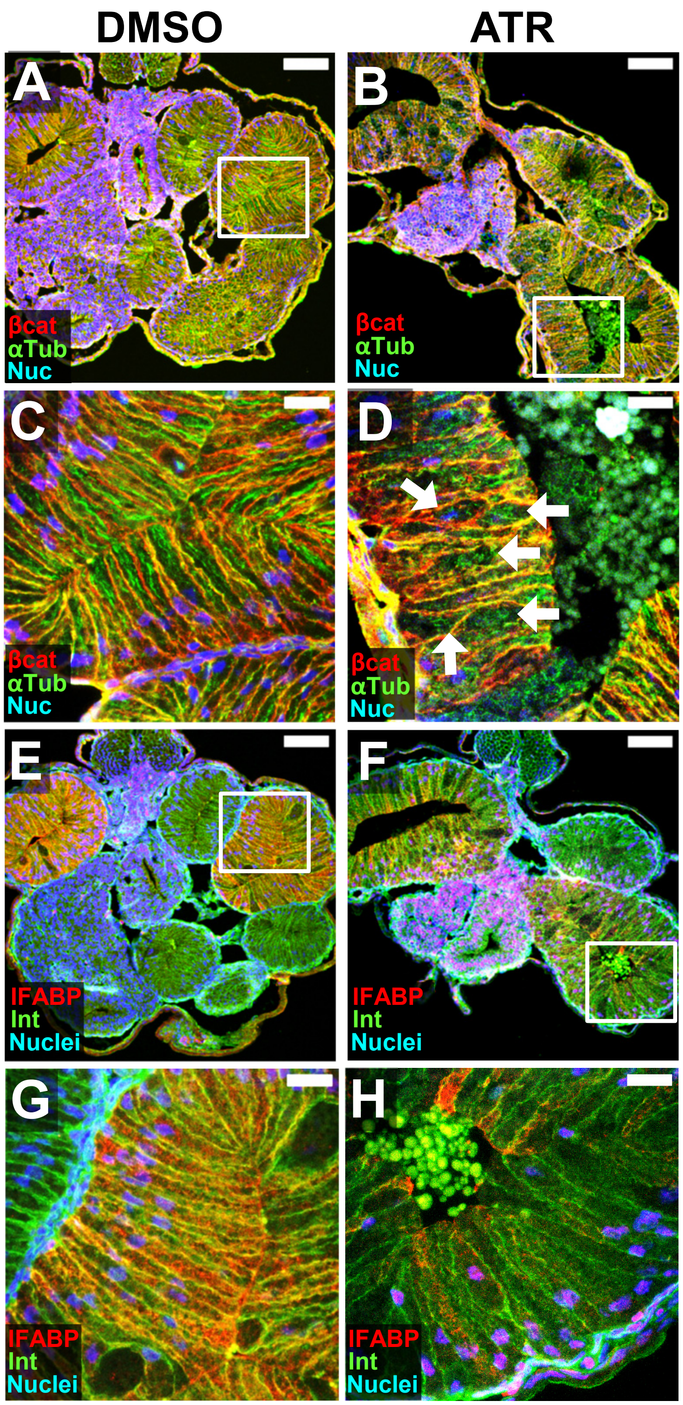

Fig. S3. ATR exposure causes late-stage abnormalities in epithelial architecture. Transverse sections of NF 44 embryos exposed to DMSO (A,C,E,G) or ATR (B,D,F,H) were immunostained for β-catenin (βcat, red; A-D), α-Tubulin (αTub, green; A-D), integrin (Int, green; E-H, to outline cell membranes), and/or IFABP (red; E-H). Nuclei (Nuc, TO-PRO-3) are blue. Boxed regions in A-B and E-F are shown at higher magnification in C-D and G-H, respectively. Epithelial cells of ATR-exposed intestines appear disorganized, wider/rounder, and have aberrant apicobasal polarity (compare C and D, arrows). Robust IFABP expression is prevalent in control intestine loops at NF 44 (E,G), whereas expression is sparse in ATR-exposed intestines (F,H). Scale bars in A-B and E-F = 100 μm. Scale bars in C-D and G-H = 25μm.

Development • Supplementary information

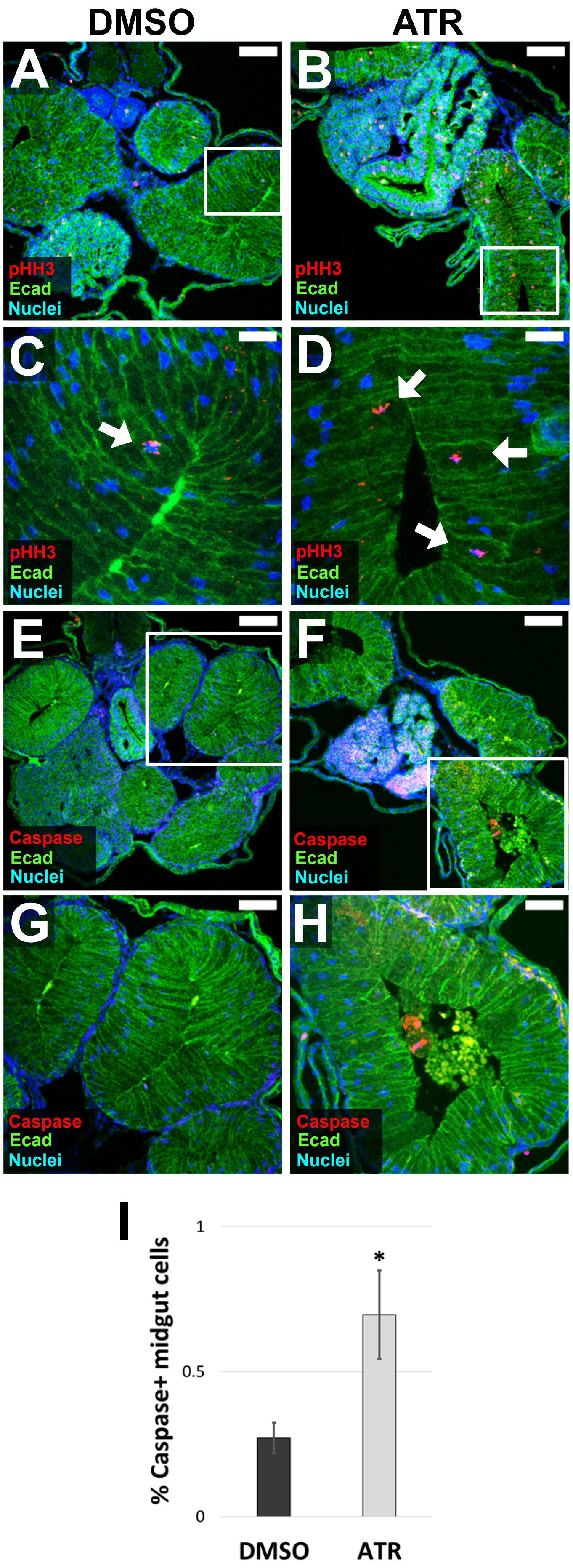

Development • Supplementary information

Fig. S4. ATR increases mitosis and apoptosis. Transverse sections through the intestine of NF 44 DMSO control and ATR-exposed embryos were immunostained for E-cadherin (Ecad, green; A-H) to visualize cell membranes, phosphohistone-H3 (pHH3, red; A-D) to identify mitotic cells, and caspase-3 to identify apoptotic cells (red; E-H). Boxed regions in A-B and E-F are shown at higher magnification in C-D and G-H, respectively. ATR-exposed intestines have a greater number of mitotic cells at the apical surface of the epithelium (compare C and D, arrows). Compared to controls (E, G) ATR-exposed intestines also have apoptotic cell debris within the lumen (F, H), as indicated by caspase staining (quantified in I). Nuclei are blue (TO-PRO-3). Error bars represent SE. *$p<0.05$. Scale bars in A-B and E-F = 100 µm. Scale bars in C-D = 25 µm. Scale bars in G-H = 50 µm.

Negative Mode Metabolomics

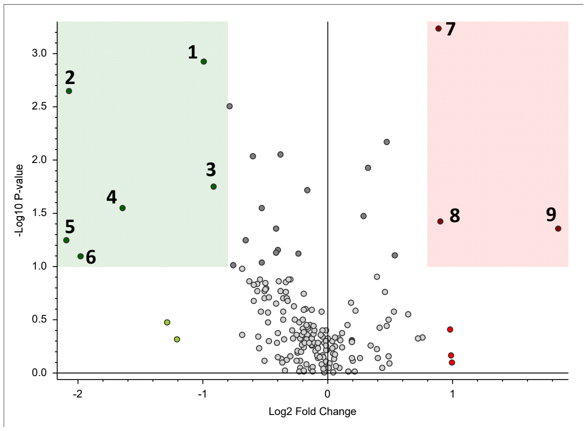

Compound Name	ID Confidence	Formula	Annot. DeltaMass [ppm]	Calc. MW	m/z	Area (Max.)
1. N-Ethylglycine	Level 3	C4 H9 N O2	-1.38	103.0632	102.0559	2.87E+06
2. Malonic acid	Level 2	C3 H4 O4	-0.73	104.0109	103.0036	3.32E+07
3. Succinate	Level 1	C4 H6 O4	-0.64	118.0265	117.0193	1.76E+07
4. Xanthine	Level 3	C5 H4 N4 O2	-0.92	152.0333	151.0260	1.34E+07
5. D-Glucose 6-phosphate	Level 2	C6 H13 O9 P	-0.02	260.0297	259.0224	1.91E+07
6. β-D-Fructose 6-phosphate	Level 2	C6 H13 O9 P	-0.09	260.0297	259.0224	1.00E+07
7. Aceglutamide	Level 2	C7 H12 N2 O4	-0.34	188.0796	187.0724	3.91E+07
8. Arabic acid	Level 3	C5 H10 O6	-0.65	166.0476	165.0404	9.29E+07
9. N-Acetyl-L-tyrosine	Level 2	C11 H13 N O4	0.12	223.0845	222.0772	1.67E+07

Positive Mode Metabolomics

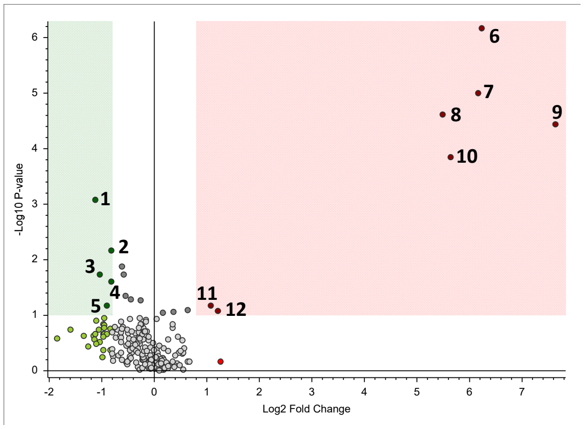

Compound Name	ID Confidence	Formula	Annot. DeltaMass [ppm]	Calc. MW	m/z	Area (Max.)
1. 6-Methyl[1,2,4]triazolo [4,3-b]pyridazin-8-ol	Level 3	C6 H6 N4 O	-0.55	150.0541	151.0614	5.46E+06
2. 5-Amino-1,2-oxazol-3(2H)-one	Level 3	C3 H4 N2 O2	-1.02	100.0272	118.0610	2.18E+07
3. HEPES	Level 2	C8 H18 N2 O4 S	-0.67	238.0986	239.1058	6.71E+08
4. Ile-Phe	Level 3	C15 H22 N2 O3	0.36	278.1631	279.1704	1.61E+07
5. Valylproline	Level 2	C10 H18 N2 O3	-0.08	214.1317	215.1390	1.78E+07
6. Desmetryn	Level 3	C8 H15 N5 S	0	213.1048	214.1121	1.32E+07
7. Desisopropylatrazine	Level 2	C5 H8 Cl N5	-0.36	173.0468	174.0540	1.75E+07
8. Atrazine	Level 2	C8 H14 Cl N5	-0.4	215.0937	216.1010	1.24E+09
9. 2-Hydroxyatrazine	Level 2	C8 H15 N5 O	-0.01	197.1277	198.1349	2.93E+07
10. Propazine	Level 2	C9 H16 Cl N5	-0.1	229.1094	230.1167	8.34E+06
11. Pantothenic acid	Level 2	C9 H17 N O5	-0.28	219.1106	220.1179	4.58E+07
12. Stearoyl-L-carnitine	Level 2	C25 H49 N O4	-0.43	427.366	428.3732	1.05E+07

Fig. S5. Metabolite changes in intestines exposed to ATR. Volcano plots show the statistically-significant altered compounds (shaded area: p-value ≤ 0.1 and Log_2 FC ≥ 0.8 or ≤ -0.8) in the ATR-treated group vs the DMSO-control group. Dots represent features that were downregulated (green), upregulated (red) or had no observed difference (gray). Data were filtered to remove features with Pooled QC Areas >30% RSD, Annotation ΔMass outside ±2 ppm and no MS^2-based annotations. Corresponding tables list the significantly altered compounds annotated at confidence levels 1-3 as described by Schymanski et al (2014). Level 1 represents the highest confidence.

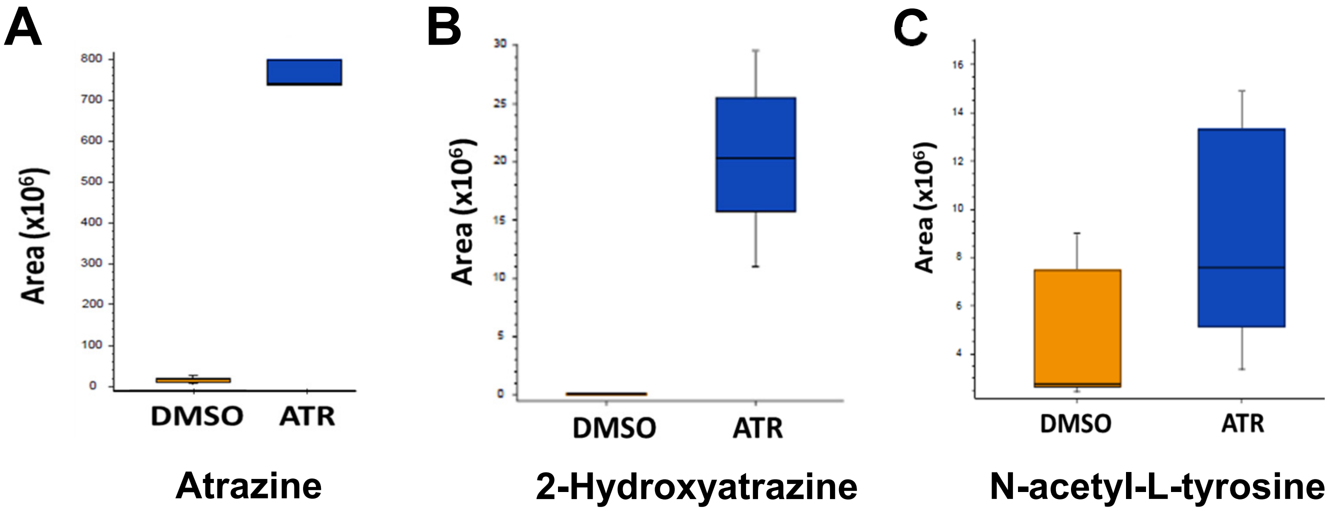

Fig. S6. Changes in select metabolites after ATR exposure. Metabolomic analysis of intestines exposed to DMSO or ATR revealed increased levels of atrazine (A), the ATR metabolite, 2-hydroxyatrazine (B), and N-acetyl-L-tyrosine (C), a metabolite associated with high ROS levels. P-value threshold: 0.05 and log2 fold change: 1.

Development • Supplementary information

Development • Supplementary information

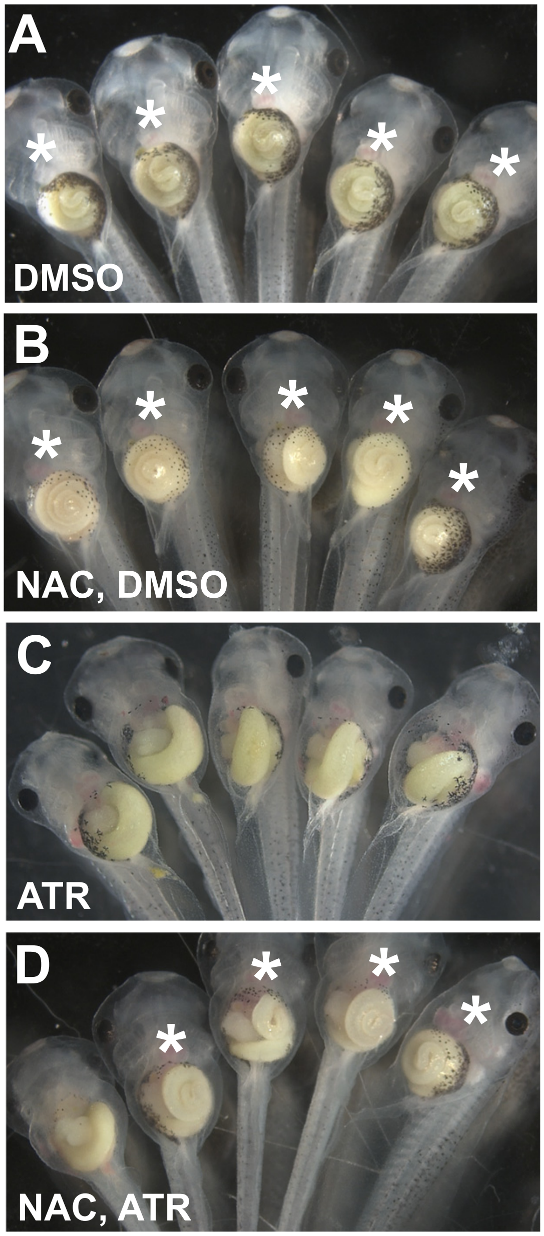

Fig. S7. Antioxidant pretreatment rescues ATR-induced intestinal elongation and rotation defects. B) Pretreating embryos with an antioxidant (NAC; B) does not affect the normal gut morphology (indicated by *) of DMSO controls (A). However, the elongation and rotation defects caused by ATR (C) are partially rescued by NAC pretreatment (D; $p<0.01$). A rescue was scored if guts exhibited 2+ intestinal coils with normal (CCW) rotation. Results were replicated in at least three independent experiments with 8-15 embryos each.

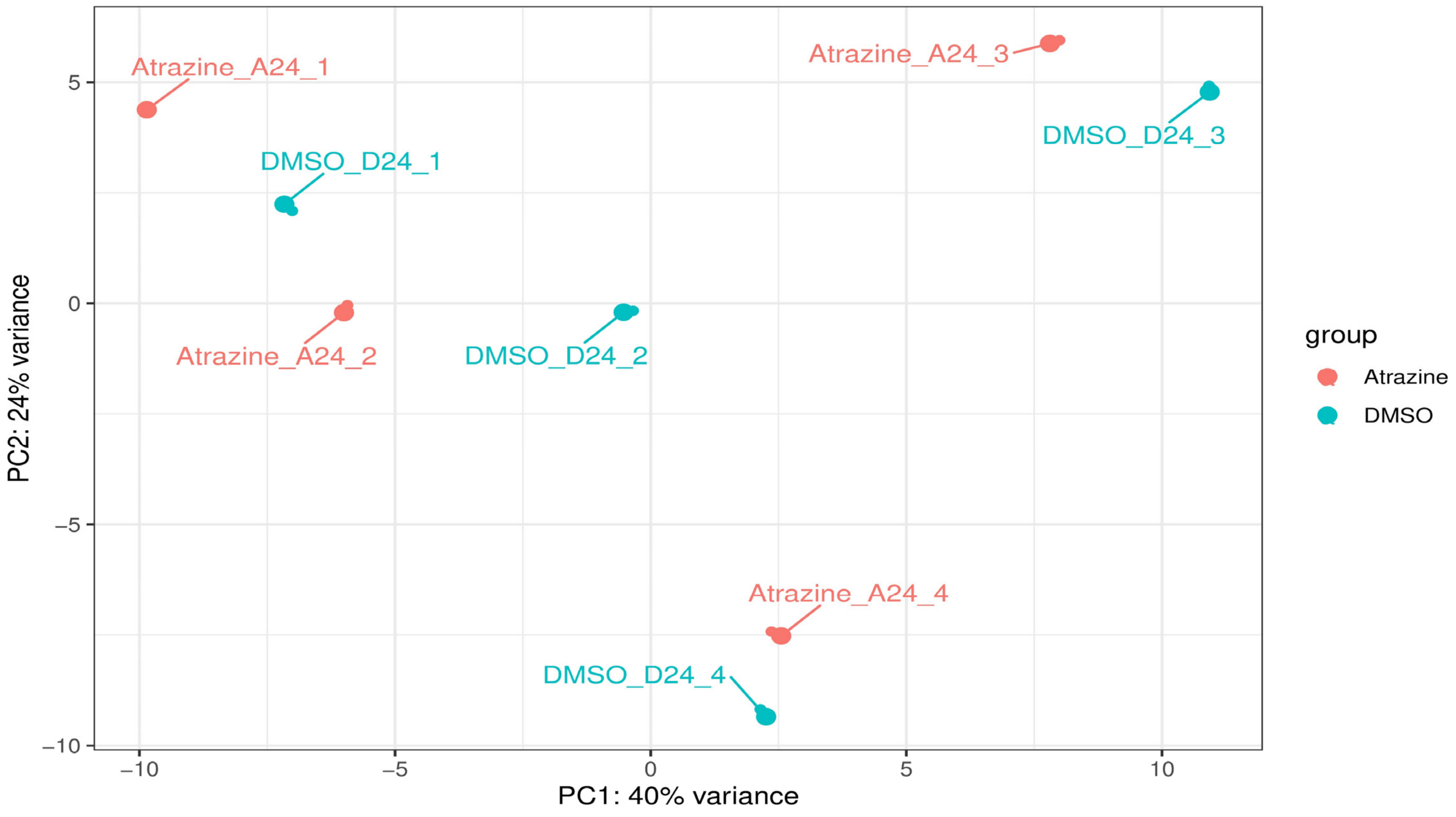

Fig. S8. Principal component analysis (PCA) of DMSO vs ATR RNAseq data. A PCA plot was generated to plot to assess sample outliers using the top 500 most variable genes after regularized log transformation.

Table S1. List of genes differentially expressed in guts exposed to ATR

Available for download at
https://journals.biologists.com/dev/article-lookup/doi/10.1242/dev.202020#supplementary-data

Development • Supplementary information
